# Subcutaneous phaeohyphomycosis caused by *Hongkongmyces snookiorum* in a kidney transplant patient: a case report

**DOI:** 10.1186/s12879-020-05295-x

**Published:** 2020-08-01

**Authors:** Deng Linqiang, Chen Yiguo, Xu Heping, Chen Dongke, Hu Longhua, Gui Xiaomei, Zou Xia

**Affiliations:** 1 Medical Laboratory, Jiangxi Provincial People’s Hospital Affiliated to Nanchang University, Nanchang, 330006 China; 2 Medical Laboratory, the First Affiliated Hospital of Xiamen University, Xianen, 361003 China; 3 Department of Laboratory Medicine, Beijing Hospital, National Center of Gerontology, Beijing, 100730 China; 4 Medical Laboratory, the Second Affiliated Hospital of Nanchang University, Nanchang, 330006 China

**Keywords:** Kidney transplant; subcutaneous infection; *Hongkongmyces snookiorum*

## Abstract

**Background:**

Morbidity and mortality in transplant patients is increased by infection caused mainly by rare opportunistic pathogens. The present study reports a case where *Hongkongmyces snookiorum* caused subcutaneous phaeohyphomycosis in a kidney transplant patient.

**Case presentation:**

A 47-year old Chinese woman with chronic kidney disease 5 underwent kidney transplantation 3 years ago. Her regular medications included Tacrolimus (1 mg, two times daily), Mycophenolate Mofetil (two times 250 mg, twice daily) and Prednisone acetate tablets (5 mg daily). Eighteen months ago, her proximal right index finger was red, painful and swollen. After admission, a hard and fluctuating 1 cm × 1 cm abscess was found on the dorsal side of the right index finger. Gram and fluorescence staining of a direct smear of a syringe extraction from the abscess revealed presence of filamentous fungi. White velvet colonies (2–3 mm) were found on blood plate and Sabouraud glucose agar (SGA) after 1 week, and grey aerial hyphae were observed. After 15 days, a 26 mm gray colony was also observed on SGA. The homology between this filamentous fungus and *Hongkongmyces snookiorum* ILLS00125755 (Genbank Sequence ID: MH161189.1) was 99.66%. An in vitro antifungal susceptibility test showed that this filamentous fungus was sensitive to azoles such as itraconazole and voriconazole.

**Conclusions:**

We report an opportunistic fungus infection caused by *Hongkongmyces snookiorum* in a transplant patient. Our finding shows that prevention of subcutaneous fungal infection is necessary for kidney transplantation patients.

## Background

Infection is an important cause of morbidity and mortality in transplant patients [1, 2]. These infections are typically caused by rare opportunistic pathogens [3–5]. Phaeohyphomycosis is caused by a large heterogeneous group of dematiaceous, or darkly pigmented, fungi. These infections are being increasingly observed in transplant patients [6]. We report for the first time a case of subcutaneous phaeohyphomycosis caused by *Hongkongmyces snookiorum* in a kidney transplant patient.

## Case presentation

**Fig. 1:**
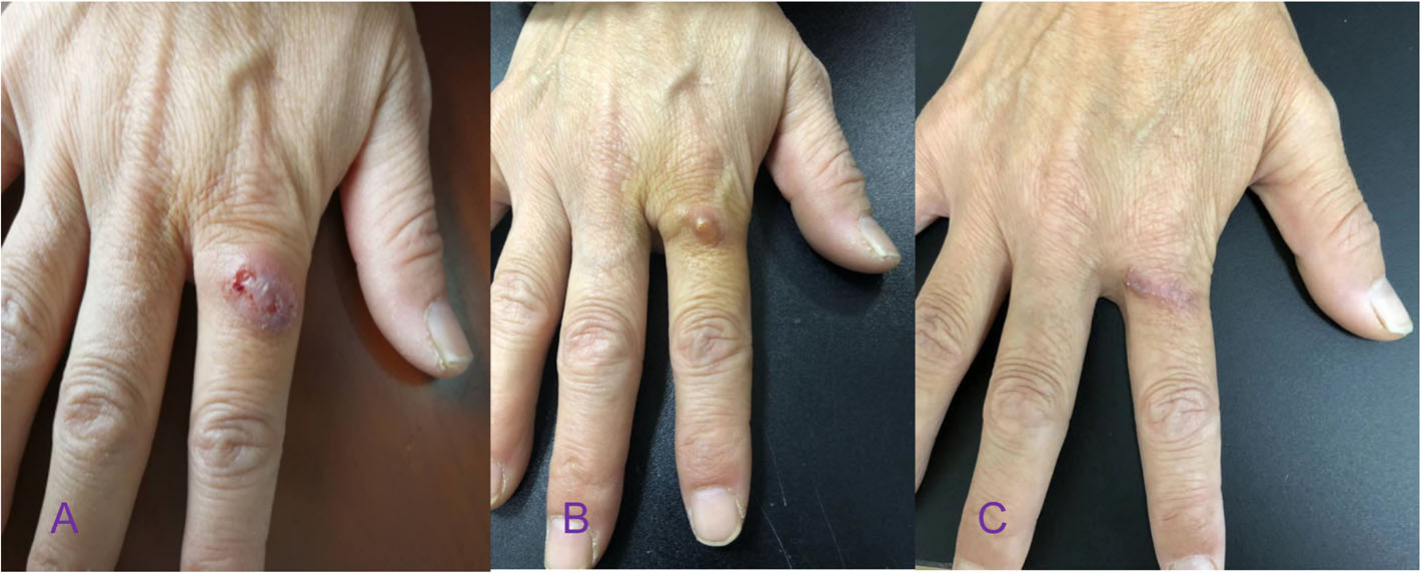
**a** Lesion at the time of initial diagnosis, (**b**) 4 weeks after the first withdrawal, (**c**) after treatment was continued for a second time

A 47-year old Chinese woman with chronic kidney disease 5 underwent kidney transplantation 3 years ago. Her regular medications included Tacrolimus (1 mg, two times daily), Mycophenolate Mofetil (two times 250 mg, twice daily) and Prednisone acetate tablets (5 mg daily). Eighteen months ago, her proximal right index finger was red, painful and swollen, with some white pus. After admission, a hard and fluctuating 1 cm × 1 cm abscess was found on the dorsal side of the right index finger (Fig. 1a). Physical examination showed that the patient was in good general condition. The transplanted kidney was tough, inactive, with no tenderness. No snoring pain, no sputum pain and no negative shifting dullness in the renal area were detected. Digital Radiography (DR) showed soft tissue swelling around the right finger joint and an increased density shadow. Laboratory tests showed that C-Reactive Protein (CRP), blood, urine, stool and coagulation functions were normal, with creatinine 141 μmol / L, urea nitrogen 9.4 mmol / L and slightly increased uric acid, 423 μmol / L.

**Fig. 2:**
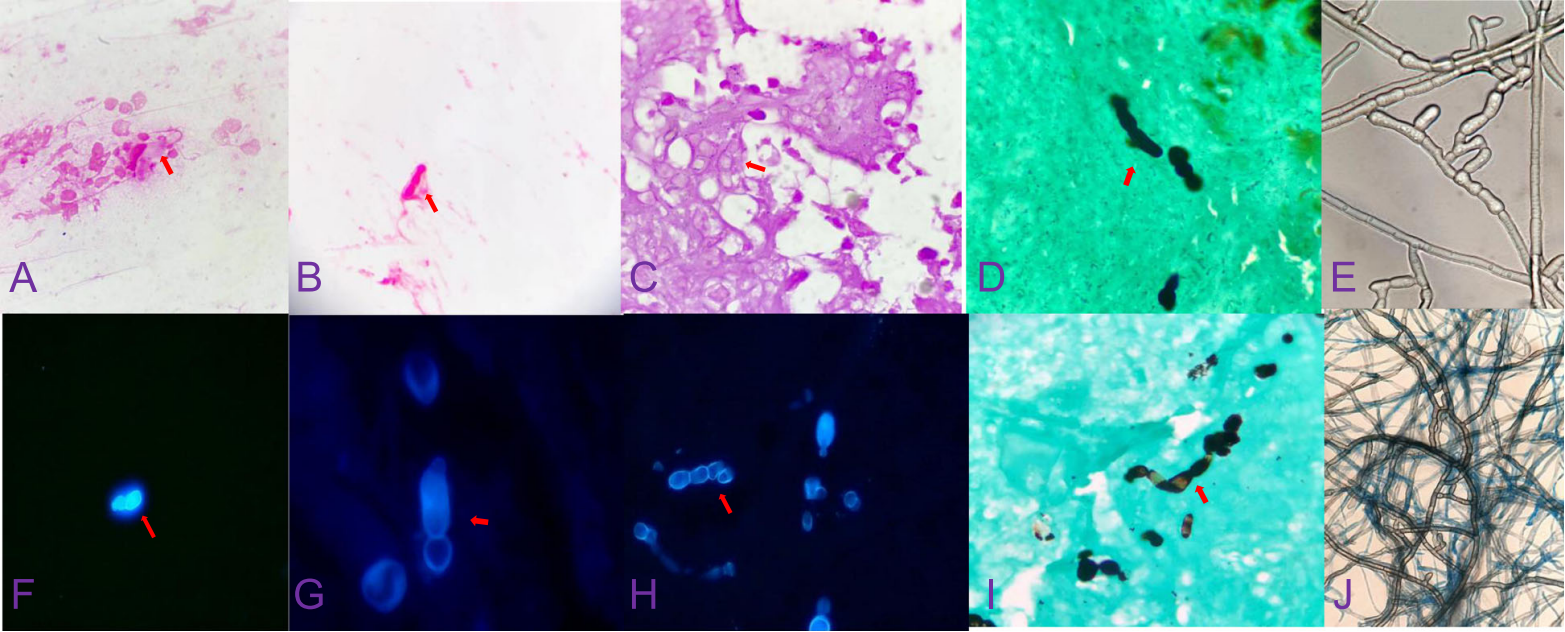
Microscopic photographs (see arrow point) corresponding to specimens of (**a**) newly diagnosed pus (Gram staining, 1000x), (**b**) newly diagnosed pathological biopsy (Gram staining, 1000x), (**c**) pathological biopsy (Gram staining, 1000x), (**d**) newly diagnosed pathological biopsy (Grocott’s methenamine silver stain, 1000x), (**e**) PDA (small culture) cultured for 6 days at 28 °C (original magnification, 1000x), (**f**) first diagnosed pus (fluorescence staining, 1000x), (**g**) first diagnosed pathological biopsy (fluorescence staining, 1000x), (**h**) pathological biopsy 4 weeks after the first withdrawal of drugs (Fluorescence staining, 1000x), (**i**) pathological biopsy 4 weeks after the first withdrawal (Grocott’s methen-amine silver stain, 1000x), (**j**) PDA (small culture) bacteria cultured at 28 °C for 12 days (lactophenol cotton blue stain, original magnification, 1000x)

Gram and fluorescence staining of a direct smear from a syringe extraction of the abscess revealed the presence of filamentous fungi (Fig. 2a and f). On the second day, the same filamentous fungus was found on the pathological tissues after crushing when the lesion was stained with Gram stain, Grocott’s methenamine silver stain and fluorescent staining (Fig. 2b, d, g).

**Fig. 3:**
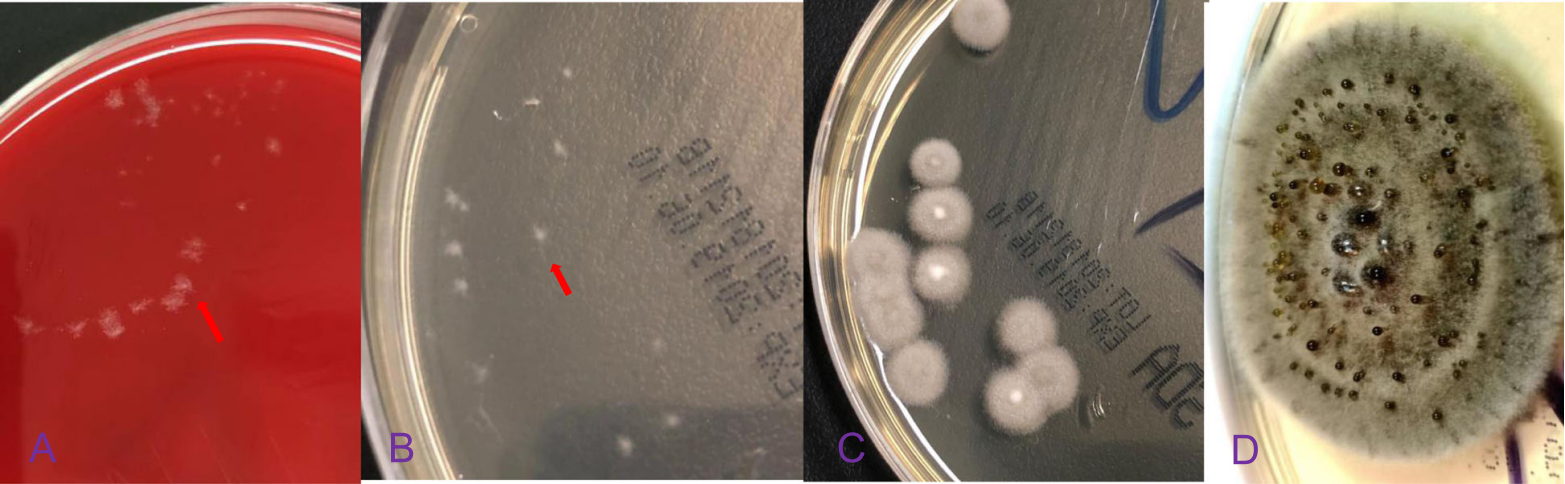
Fungal cultures. **a** Colonies cultured for 3 days at 37 °C in a blood plate, (**b**) colonies cultured for 3 days at 37 °C in SGA, (**c**) colonies cultured at 37 °C SGA for 1 week, (**d**) colonies cultured at 28 °C PDA for 15 days

When the lesion was cultured at 37 °C or 28 °C for 3 days, filamentous fungi were observed, but no bacteria. White velvet colonies (2–3 mm) were found on a blood plate and in Sabouraud glucose agar (SGA) (Fig. 3a, b). After 1 week, grey aerial hyphae were observed (Fig. 3c). After 15 days, a 26 mm villous gray colony was observed on SGA and many “dew beads”-like exudates were found in the upper layer in the middle of the colony (Fig. 3d). Potato dextrose agar (PDA) (small culture) cultured at 28 °C for 6 days (Fig. 2e) and 12 days (Fig. 2j) showed separated hyphae but no spores. The mycelium color gradually darkened, which suggested presence of a dark fungus.

In order to identify the final pathogen, we performed DNA (Internal Transcribed Spacer, ITS) gene sequencing and compared the sequence with NCBI and Myo-bank databases. ITS gene primers were synthesized by Guangzhou Aiji Bio Company (Guangzhou, china) as follows: forward, ITS-1 5′-TCCGTAGGTGAACCTGCGG-3′, reverse: ITS-4 5′-TCCTCCGCTTATTGATATGC-3′. Amplification system (25 μL): 2x Taq PCR MasterMix 12.5 μL, double distilled water 8.5 μL, 1 μL of primers, 2 μL DNA template; amplification procedure: 95 °C (5 min), 95 °C (1 min), 55 °C (1 min), 72 °C (1 min), 38 cycles, 72 °C for 10 min. ITS gene sequences were analyzed by sequence scanner software (version 1.0) (Guangzhou Aiji Bio Company, China) and the quality value (QV) of sequence > 20 was selected and compared in NCBI and Myo-bank databases. The identification results were analyzed by the guide of CLSI MM18-A. We found that the homology between this filamentous fungus and *Hongkongmyces snookiorum* ILLS00125755 (Genbank Sequence ID: MH161189.1) was 99.66%.

The susceptibility of *Hongkongmyces snookiorum* to three antifungal drugs (amphotericin B, voriconazole and itraconazole) was tested using an E-test method (Trek Diagnostic Systems, East Grinstead, UK), performed according to the manufacturer’s protocol for filamentous fungi. Briefly, *Hongkongmyces snookiorum* was cultured at 28 °C for 7 days. The concentration of hyphal fragments was adjusted to 1.0 McFarland standard (refer to CLSI M38-2A). A sterile cotton swab was immersed in the adjusted bacterial suspension. Excessive bacterial suspension was extruded on the wall of the tube, and streaked on the fungal drug sensitive agar plate. After 3–5 min at room temperature, E-test (Zhengzhou Antu Bio, China) was added to the plate and incubated at 35 °C for 7 days. The minimum inhibitory concentration in the E-test was obtained following manufacturer’s instructions. The test was performed in duplicate. The strain used for quality control was *Candida albicans* ATCC90028. The antifungal susceptibility test (E-test) showed sensitivity to azoles such as itraconazole and voriconazole, with MIC of 0.012 and 0.008 μg/mL, respectively, although resistance was observed against amphotericin B (MIC: 4 μg/ml). Administration of oral voriconazole (100 mg twice daily) combined with an operation resection was effective in the treatment of the lesion.

## Discussion and conclusions

Phaeohyphomycosis is caused by a large, heterogenous group of dematiaceous, or darkly pigmented fungi. *Hongkongmyces snookiorum* belongs to the fungal family *Lindgomycetaceae,* in the order Pleosporales within the class Dothideomycetes*. Hongkongmyces snookiorum* has been found only in Pennsylvania, USA, isolated from submerged detritus from a fresh water fen. Among its sister taxa, only *Hongkongmyces pedis* was associated to human infections previously [7–13].

Our results show that the isolated fugus can produce mycelium but no spores when cultured on SGA and PDA for 30 days at 28 °C and 37 °C, respectively. The fungus was not identified by Matrix Assisted Laser Desorption Ionization - Time of Flight (MALDI-TOF) (bioMérieux, France and Jiangsu Skyray Instrument, China) probably because its limited presence in databases [14, 15]. When identified by DNA sequencing, its ITS1 sequence was longer and more variable than in related members of the Lindgomycetaceae family (about 500 bp) [16]. We further sequenced the ITS gene of the fungus using universal primers ITS-1 and ITS-4, and found that the homology between this isolated fungus and *Hongkongmyces snookiorum* ILLS00125755 (Genbank Sequence ID: MH161189.1) is 99.66%.

The patient is a 47-year-old woman with a kidney transplant that was exposed to long-term use of anti-rejection and immunosuppressive agents and hormones, such as tacrolimus, mycophenolate mofetil and prednisone acetate. These risk factors may be causing opportunistic fungal infections. As a farmer and a housewife, the patient has lived in a hilly region of southern China for a long time and may be exposed to such fungi.

Invasive skin and subcutaneous infections caused by demalaceous fungi are transmitted through wounds, which are often neglected by patients. The patient could not describe exactly the cause of the wound or when it appeared. The same hypha was found in smear and cultures of pus specimens and biopsies from the patient. Combination of local septic granule inflammatory, those evidences above suggested the patient did have a *Hongkongmyces snookiorum* infection.

After the patient was discharged from the hospital, the infection symptoms were controlled by administration of oral voriconazole, 100 mg twice a day for 4 weeks. However, when the patient stopped taking the drug, the lesion pain and ulceration reappeared after 2 weeks at the same site, with several small nodules around it. The size was approximately 0.3 cm × 0.2 cm × 0.1 cm (Fig. 1b), and histopathological features were similar to previous results (Fig. 2c, h, i). The patient was again given voriconazole orally, 100 mg twice daily, and the condition improved significantly after 4 weeks (Fig. 1c). No recurrence was found after a ten-month follow-up.

Thus, kidney transplantation patients must pay attention to prevent subcutaneous fungal infection.

## Data Availability

All data generated or analyzed during this study are included in this published article.

## Abbreviations

SGA: Sabouraud glucose agar
PDA: Potato dextrose agar;
DR: Digital Radiography
CRP: C-Reactive Protein
DNA: Deoxyribonucleic acid
ITS: Internal Transcribed Spacer gene
MIC: Minimal inhibitory concentration
MALDI-TOF: Matrix Assisted Laser Desorption Ionization - Time of Flight
